# Effect of Tadalafil on cardiac function and left ventricular dimensions in Duchenne muscular dystrophy: safety and cardiac MRI substudy results from a randomized, placebo-controlled trial

**DOI:** 10.1186/s12872-025-04727-3

**Published:** 2025-04-11

**Authors:** David Cox, Barry Byrne, David W. Hammers, John Landry, H. Lee Sweeney

**Affiliations:** 1 Lilly Research Laboratories, Lilly Corporate Center, Eli Lilly and Company, 893 S. Delaware Street, Indianapolis, IN 46285 USA; 2 University of Florida, Gainesville, FL USA; 3 Eli Lilly Canada, Eli Lilly and Company, Toronto, ON Canada

**Keywords:** Duchenne muscular dystrophy, Tadalafil, PDE5 inhibitor, Cardiac

## Abstract

**Background:**

Inhibition of phosphodiesterase 5 (PDE5) was hypothesized to slow disease progression in Duchenne muscular dystrophy (DMD). Tadalafil, a once-daily PDE5 inhibitor, did not slow loss of ambulation in a phase 3 placebo-controlled trial. This report details the cardiac findings from this study.

**Methods:**

Patients with DMD (*N* = 331) aged 7 to 14 years on stable glucocorticoids were randomized to tadalafil 0.3 mg/kg/day, 0.6 mg/kg/day, or placebo. Ejection fraction (EF), fractional shortening, and M-mode ventricular dimensions were measured on echocardiograms. 12-lead ECGs were centrally evaluated for heart rate and intervals, and qualitative diagnoses. Vital signs and unsolicited adverse events were collected throughout the study. Cardiac MRI (CMR) was collected in a subset of 27 patients. Z-scores for ventricular dimensions and volumes were calculated based on published age-normative reference values. Treatment differences for change in continuous ECG parameters and vital signs were compared using Wilcoxon rank-sum tests. Echocardiogram and CMR parameters were analyzed with an ANCOVA model.

**Results:**

Tadalafil had no adverse effects on echocardiographic left ventricular (LV) EF or fractional shortening, ECG findings, or vital signs. Mean diastolic LV internal dimension (LVIDd) was increased in the tadalafil 0.6 mg/kg group versus placebo at Week 24 (+ 0.13 cm, *p* =.019) and Week 48 (+ 0.18 cm, *p* =.008), with a similar pattern observed for LV systolic dimensions (LVIDs). Mean LV end diastolic volume (EDV) measured by CMR also increased at Week 48 in the tadalafil 0.3 mg/kg (+ 13.0 ml, *p* =.047 vs. placebo) and 0.6 mg/kg (+ 12.0 ml, *p* =.08 vs. placebo) groups, with numerically smaller increases in LV EDV and commensurate increases in stroke volume and cardiac output. Z-scores for LVIDd and LV EDV were generally below the normal range at baseline and increased toward or within the normal range in the tadalafil groups but not in the placebo group.

**Conclusions:**

No adverse effects of tadalafil on cardiovascular function were evident based on adverse events, echocardiograms, ECG, or vital sign measurements through 48 weeks in patients with DMD. The small mean increases in LVID and LV volume observed with tadalafil are consistent with PDE5 inhibitor pharmacology, but their clinical relevance in the context of LV tonic contraction in DMD is unknown and deserve further study.

**ClinicalTrials.gov identifier:**

NCT01865084 (first registration date: 24-May-2013).

## Introduction

Duchenne muscular dystrophy (DMD) affects one in 3,500 to 5,000 live male births. Boys are diagnosed as toddlers and most lose ambulation by the age of 12 years. Death often occurs in the second decade from ventilatory insufficiency and progressive cardiac dysfunction with arrhythmia [1]. With advancements in pulmonary care and mechanical ventilation, patients with DMD are living longer such that cardiomyopathy has become the most common cause of death in this population [2].

The pathophysiology of cardiomyopathy in DMD is complex but is thought to result primarily from disruptions in calcium handling and sarcolemmal membrane integrity due to the absence of cardiac dystrophin [3]. Cardiac inflammation and fibrosis by gadolinium enhancement [4] and changes in ventricular strain measures [5] are evident in younger boys with DMD prior to onset of clinical signs of cardiac dysfunction. Although variable, declines in global measures of cardiac function such as left ventricular ejection fraction (LVEF) typically first appear later in disease progression (around 10 years of age), with most patients having clinical evidence of cardiac dysfunction and dilation by the time they are adolescents and young adults [6, 7].

The effects of PDE5 inhibition on cardiac function or progression of dystrophic cardiomyopathy remains unclear. Studies with sildenafil in the mdx mouse model support the concept that PDE5 inhibition may have favorable effects to prevent or ameliorate cardiac dysfunction associated with dystrophin deficiency [8, 9]. Prophylactic treatment of dogs with golden retriever muscular dystrophy (GRMD) with tadalafil preserved cardiac systolic function, improved measures of circumferential wall strain, and reduced histopathology of cardiac muscle compared to untreated GRMD animals [10]. On the other hand, acute (4-week) treatment of men with Becker muscular dystrophy had no detectable effect on cardiac function, and the REVERSE-DBMD trial, a sildenafil study in adults with advanced DMD (mean age, ~ 23–26 years) and existing left ventricular dysfunction, was stopped early due to a finding of increased left ventricular end systolic volume in the sildenafil group [11].

Based on promising preclinical data from multiple dystrophic animal models [8, 12–15] and proof-of-mechanism data from patients with muscular dystrophy [16], a large Phase 3 randomized placebo-controlled trial was conducted in 331 boys and young men with DMD to test the hypothesis that once-daily tadalafil, administered orally for 48 weeks, would slow the decline in ambulatory function in these patients. This trial failed to meet its primary and secondary efficacy objectives [17]. Because of the importance of monitoring cardiac function in patients with DMD, and the potential for cardiac effects of PDE5 inhibition, this trial also collected serial echocardiograms, electrocardiograms, vital signs, and adverse events as safety measures from all randomized patients and performed cardiac magnetic resonance (CMR) imaging in a subset of patients. This paper reports the detailed cardiac findings from this study.

## Methods

### Study design

Details of the study design have been published previously [17]. Briefly, this was a phase 3, global, multicenter, randomized, double-blind, placebo-controlled, parallel, 3-arm study of tadalafil in patients with DMD who were being treated with corticosteroids. Patients were recruited at 63 sites in 15 countries.

### **Selection** of study populatio**n**

Patients were eligible to be included in the study if they were male with proven DMD, between the ages of 7 to 14 years (inclusive), ambulant (defined as screening and baseline 6MWD measurements between 200 and 400 m), baseline 6MWD measurements within 20% of the screening 6MWD, left ventricular ejection fraction (LVEF) ≥ 50%, and received systemic corticosteroids for a minimum of 6 months immediately prior to screening. Key exclusion criteria included patients who had symptomatic cardiomyopathy or heart failure, or cardiac rhythm disorder defined as sinus rhythm with ectopic contractions or conductance disturbances, or any rhythm other than sinus, observed on screening ECG, use of continuous mechanical ventilator assistance, history of renal insufficiency, severe hypotension or uncontrolled hypertension, or current treatment with potent CYP3A4 inhibitors. Prophylactic treatment for heart failure was allowed but should have been stable with no changes (initiation, change in type of drug, dose modification, schedule modification, interruption, discontinuation, or reinitiating) within 3 months prior to the start of study treatment.

### Randomization and blinding

Eligible patients were randomly assigned using an interactive voice-response/web-based system to 1 of 3 treatment arms (placebo, tadalafil 0.3 mg/kg, or tadalafil 0.6 mg/kg) in a 1:1:1 ratio. Randomization was stratified by site and baseline 6MWD (< 300 m versus ≥ 300 m). Regardless of treatment assignment and dose, all patients received the same number of tablets to maintain blinding. Patients, investigators, and other study staff were blinded to treatment assignment.

### Procedures

An echocardiogram was obtained at screening to confirm the patient’s LVEF met the entry requirement (≥ 50%) unless a record of an echocardiogram obtained within 30 days of Visit 1 was obtained confirming the patient met this criterion. Follow-up echocardiograms were obtained from all patients at Week 24 and Week 48. Echocardiograms were performed per local standard of care, but were to include, at a minimum, 2 dimensional and M-mode imaging, and were to be performed without contrast. Echocardiographic measures included the LVEF (measured from 2D echocardiography based on ventricular volumes using modified Simpson method) and left ventricular internal diameters at diastole (LVIDd) and systole (LVIDs) were measured as linear dimensions from M-mode echocardiography. No central reading of echocardiograms was performed. Fractional shortening was calculated as (LVIDs-LVIDd/LVIDd) x 100.

Serial 12-lead digital ECGs were collected locally at baseline, Week 24, and Week 48 (or early termination). ECGs were interpreted by the investigator or qualified designee at the site as soon after the time of ECG collection as possible, and ideally while the patient was still present, to determine whether the patient met entry criteria and for immediate patient management, if any clinically relevant findings were identified. All digital ECGs were electronically transmitted to a designated central ECG laboratory. A cardiologist at the central ECG laboratory conducted a full overread on the ECG (including all intervals). A report based on data from this analysis was issued to the investigative site. All data from the overreads were placed in the database for analytical and reporting purposes.

CMR imaging was collected as an exploratory sub-study performed at sites in the US with the appropriate equipment and experience to perform the procedure. CMR was performed without contrast or sedation at baseline and Week 48 (or endpoint). Sites could use the imaging protocol that was standard of care at their facility, as long as it included the imaging sequences required for central assessment of functional parameters. All CMR images were transferred to a central imaging laboratory (Biomedical Systems). A technical expert at the central imaging laboratory conducted a full quality review of each CMR image and collected functional and morphological measures including LVEF, EDV and ESV, LV mass, cardiac output, stroke volume, and global circumferential wall strain.

Blood pressure and heart rate were measured at each study visit after the patient had been in a supine position for at least 2 min, using the same arm for each measurement. Adverse events were collected in an unsolicited manner at each study visit and subsequently coded to MedDRA (Version 18.1).

### Z-score calculations

Results published after primary database lock for the study suggested that the heart in younger boys with DMD is characterized by smaller-than-normal left ventricular size detectable on echocardiograms as LV internal dimensions that are smaller than age-normative expected values particularly between the ages of 11 and 15 years [18]. Therefore, post-hoc analyses were performed to calculate Z-scores for echocardiogram parameters of LV internal dimensions based on algorithms developed by Boston Children’s Hospital and provided by Dr. Steve Colan (https://zscore.chboston.org/). Z-scores corrected for body surface area (BSA) for cMRI-measured ventricular volumes were calculated based on comparison to pediatric normative values for boys 8–15 years of age [19]. Data for LV volumes and dimensions were normalized for BSA to adjust for changes in body size and a Z-score was calculated for each value as a measure of deviation (number of SDs) from the expected mean normative value.

### **Retrospective** analysis canine echocardiogram data

Prior work by two of the coauthors (HLS and DWH) demonstrated that daily administration of tadalafil to dogs with muscular dystrophy prior to the onset of cardiac functional decline preserved LV function as measured by echocardiography and improved GRMD cardiac histopathology [10]. Considering the clinical findings reported herein, a retrospective analysis of the echocardiogram data from this animal study was performed, with the addition of 3 GRMD control data sets acquired since the publication of the initial study (total of 5 control and 2 tadalafil-treated). Echocardiography was performed pre-treatment (taken at 6 months of age) and post-tadalafil treatment (taken at 18 months of age) using a Philips CX-50 system (Philips) and an 8‐ to 3‐MHz transducer as described previously [10, 20]. In treated animals, tadalafil was administered orally (1 mg/kg) daily beginning at 9 months of age.

### Statistical methods

Change from baseline to endpoint treatment differences in continuous ECG parameters were assessed using Wilcoxon rank-sum tests. The number and percentage of patients with treatment-emergent abnormal qualitative ECG (central read) results at any time were summarized by treatment group and compared between treatments using Fisher’s exact tests of each tadalafil group versus placebo. Change from baseline to endpoint treatment differences in vital signs were assessed pairwise using Wilcoxon rank-sum tests. Echocardiogram parameters collected at the 24- and 48-week visits were analyzed through an ANCOVA model at each visit with treatment and site as factors and baseline as a covariate. Changes from baseline to last observation for each cardiac MRI parameter were analyzed using an ANCOVA model with change from baseline value as the dependent variable, baseline value as the covariate, and treatment included as an independent fixed effect.

## Results

### Patient characteristics

Detailed baseline demographics and characteristics of patients were reported previously [17]. The overall mean age was 9.6 years, and the majority of patients were White (79.2%). Vital signs and echocardiography measures were generally balanced across treatment groups at baseline (Table 1). The most common concomitant cardiac medication reported during the trial was ACE inhibitors, with a slightly higher proportion of patients in the tadalafil 0.3 mg/kg group reporting use of an ACE inhibitor (26.5%) than those in the tadalafil 0.6 mg/kg or placebo groups. All randomized patients were taking a corticosteroid at baseline: 53.8% were taking prednisone/prednisolone and 45.9% were taking deflazacort. The mean duration of corticosteroid therapy (from first use) was 40.6 months. Some numerical imbalances across treatment groups in baseline CMR parameters were observed, most notably a smaller LV end diastolic volume (EDV), LV stroke volume, and LV mass in the tadalafil 0.3 mg/kg group (Table 1).

**Table 1:** Patient characteristics

	Placebo (*N* = 116)	Tadalafil 0.3 mg/kg (*N* = 102)	Tadalafil 0.6 mg/kg (*N* = 113)
Age, years	9.4 (1.76)	9.9 (2.26)	9.5 (1.71)
BMI, kg/m2	19.7 (4.45)	20.2 (4.26)	19.6 (4.79)
Vital signs			
Systolic blood pressure, mmHg	107.2 (10.7)	108.6 (10.6)	109.9 (11.3)
Diastolic blood pressure, mmHg	66.7 (10.2)	65.8 (10.7)	66.0 (10.7)
Heart rate, beats per minute	94.8 (11.7)	96.1 (12.2)	93.5 (13.7)
Echocardiography parameters			
LVEF, %	63.5 (6.4)	63.2 (5.6)	62.4 (6.8)
LV shortening fraction, %	35.0 (6.3)	35.5 (5.2)	34.7 (5.5)
LVIDd, cm	3.96 (0.39)	4.00 (0.42)	4.02 (0.40)
LVIDs, cm	2.58 (0.39)	2.59 (0.36)	2.63 (0.36)
Cardiac medications, n (%)			
ACE inhibitors	20 (17.2)	27 (26.5)	20 (17.7)
Ubiquinone	14 (12.1)	10 (9.8)	8 (7.1)
Angiotensin II antagonists	5 (4.3)	3 (2.9)	3 (2.7)
Beta-blocking agents	2 (1.7)	3 (2.9)	1 (0.9)
CMR parameters *(Substudy* ***N*** *= 27)*	**Placebo** **(** ***N*** **= 9)**	**Tadalafil 0.3 mg/kg (** ***N*** ** = 10)**	**Tadalafil 0.6 mg/kg (** ***N*** ** = 8)**
LVEF, %	59.8 (5.2)	59.6 (5.0)	59.2 (2.2)
Circumferential strain, %	-23.2 (3.5)	-22.1 (2.3)	-22.8 (9.8)
LV end diastolic volume, mL	74.3 (19.2)	68.7 (12.4)	77.3 (31.9)
LV end systolic volume, mL	29.5 (6.4)	28.2 (8.1)	31.9 (14.8)
LV stroke volume, mL	44.8 (13.8)	40.5 (5.0)	45.4 (17.1)
Cardiac output, L/min	4.2 (1.1)	4.1 (0.7)	4.6 (1.2)
LV mass, g	44.5 (12.9)	39.0 (7.8)	46.5 (17.0)

Unless noted otherwise, all data are expressed as mean (standard deviation)

### Electrocardiogram parameters

Baseline ECG abnormalities were common: 43.3% of patients had conduction abnormalities and 27.9% had rhythm abnormalities at baseline (primarily normal sinus rhythm with sinus arrhythmia). There were no treatment group differences in the proportion of patients with an overall treatment-emergent qualitative ECG assessed by the central reader as being clinically abnormal: 13.8% in placebo, 8.8% in tadalafil 0.3 mg/kg (*p* =.291), and 7.1% in tadalafil 0.6 mg/kg (*p* =.131). No patients had ECG changes indicating myocardial ischemia, injury, or infarction during the study.

Consistent with expectations, baseline mean ECG-measured heart rate was high (93–94 bpm) in this study population relative to boys without DMD. Mean decrease in heart rate was numerically larger in the tadalafil 0.3 mg/kg group (-3.3 bpm, *p* =.273) and 0.6 mg/kg group (-3.1 bpm, *p* =.366) compared to the placebo group, with corresponding numerical mean increases in intervals (Table 2).

**Table 2:** Summary of quantitative ECG parameters

Measure	Placebo (*n* = 116)		Tadalafil 0.3 mg/kg (*n* = 102)		Tadalafil 0.6 mg/kg (*n* = 112)	
	Mean Baseline^a^ (SD)	Mean Change at Endpoint^b^ (SD)	Mean Baseline^a^ (SD)	Mean Change at Endpoint^b^ (SD) *p*-value^c^	Mean Baseline^a^ (SD)	Mean Change at Endpoint^b^ (SD) *p*-value^c^
Heart rate, beats/min	93.3 (12.2)	-0.8 (12.9)	93.6 (12.0)	-3.3 (13.7) *p* =.273	94.2 (12.4)	-3.1 (12.5) *p* =.366
QT interval, msec	344.3 (22.2)	1.4 (18.5)	345.5 (23.2)	4.8 (22.1) *p* =.319	342.6 (23.9)	6.5 (19.8) *p* =.182
QTcF, msec	398.1 (16.4)	0.2 (14.5)	399.9 (17.5)	0.4 (13.4) *p* =.816	397.2 (17.5)	3.1 (13.9) *p* =.300
RR interval, msec	650.5 (91.2)	7.5 (91.7)	648.5 (90.9)	23.9 (104.2) *p* =.247	644.5 (86.7)	22.5 (88.1) *p* =.337
PR interval, msec	120.4 (12.3)	0.1 (8.0)	117.6 (11.0)	1.0 (7.7) *p* =.263	120.1 (14.5)	1.3 (7.8) *p* =.130
QRS interval, msec	82.9 (7.1)	0.7 (4.4)	82.1 (7.5)	1.6 (4.2) *p* =.128	82.8 (7.1)	1.0 (4.9) *p* =.930

^a^Baseline = the last non-missing value before first dose

^b^Endpoint = the last non-missing postbaseline value

^c^The p-values for the comparison in the difference between placebo and active treatment are obtained using Wilcoxon rank-sum tests

### **Vital** signs and cardiac-related adverse events

Mean changes in vital signs from baseline to each study visit and endpoint are summarized in Table 3. There were no clinically notable treatment group differences. The small mean decrease in ECG-measured heart rate was not apparent in the vital sign pulse rate data.

**Table 3:** Summary of vital sign

Mean change from baseline^a^ (SD) in vital sign	Placebo (*N* = 116)	Tadalafil 0.3 mg/kg (*N* = 102)	*p*-value^c^	Tadalafil 0.6 mg/kg (*N* = 112)	*p*-value^c^
**Systolic blood pressure**,** mmHg**					
Week 4	-0.17 (11.2)	0.34 (10.9)	*0.495*	-3.21 (10.0)	***0.024***
Week 12	1.18 (11.8)	-2.12 (10.4)	*0.087*	-1.60 (10.9)	*0.234*
Week 24	1.62 (11.2)	-2.03 (10.4)	***0.011***	-0.77 (11.9)	*0.106*
Week 36	1.96 (11.0)	0.82 (10.4)	*0.682*	-1.94 (11.7)	***0.023***
Week 48	1.04 (11.0)	0.29 (11.1)	*0.484*	0.50 (12.7)	*0.693*
Endpoint^b^	1.45 (11.3)	0.22 (11.0)	*0.348*	0.32 (12.5)	*0.457*
**Diastolic blood pressure**,** mmHg**					
Week 4	-2.02 (10.4)	-0.23 (10.6)	*0.616*	-2.03 (10.6)	*0.455*
Week 12	-0.42 (11.9)	-1.55 (10.1)	*0.292*	-0.03 (11.5)	*0.809*
Week 24	0.65 (9.6)	-1.94 (9.7)	***0.023***	1.26 (13.2)	*0.772*
Week 36	0.90 (10.4)	-0.97 (10.3)	*0.256*	-0.30 (11.3)	*0.397*
Week 48	0.54 (10.1)	-1.35 (11.7)	*0.232*	-0.36 (10.4)	*0.237*
Endpoint^b^	0.50 (10.1)	-1.69 (11.6)	*0.145*	-0.45 (10.2)	*0.220*
**Heart rate**,** beats per minute**					
Week 4	-0.43 (13.4)	0.89 (12.9)	*0.260*	1.98 (15.2)	*0.250*
Week 12	1.65 (14.3)	1.15 (13.5)	*0.646*	4.38 (13.6)	*0.243*
Week 24	-1.04 (13.2)	-0.49 (15.1)	*0.678*	0.42 (14.8)	*0.492*
Week 36	-0.27 (13.9)	0.90 (15.3)	*0.557*	-0.18 (14.6)	*1.000*
Week 48	-0.18 (13.5)	-0.62 (16.4)	*0.771*	-0.25 (15.2)	*0.821*
Endpoint^b^	0.15 (13.4)	-0.74 (16.3)	*0.578*	-0.56 (15.1)	*0.560*

N = number of patients in the safety analysis set.^a^Baseline = the last non-missing value before first dose

^b^Endpoint = the last non-missing postbaseline value

^c^The p-values for the comparison in the difference between placebo and active treatment are obtained using Wilcoxon rank-sum tests

Twelve patients reported TEAEs in the MedDRA System Order Class *Cardiac Disorders*: 4 (3.4%) in the placebo group, 5 (4.9%) in the tadalafil 0.3 mg/kg group, and 3 (2.7%) in the tadalafil 0.6 mg/kg group. There was no significant difference in the reporting of cardiac-related TEAEs overall or with any individual cardiac-related TEAE (Table 4). The event of myocarditis was the only cardiac-related TEAE that was a serious adverse event.

**Table 4:** Cardiac-related treatment-emergent adverse events (TEAEs)

MedDRA Preferred Term	Placebo *N* = 116 *n* (%)	Tadalafil 0.3 *N* = 102 *n* (%)	Tadalafil 0.6 *N* = 112 *n* (%)
**Subjects with > = Cardiac Disorder TEAE**	**4 (3.4)**	**5 (4.9)**	**3 (2.7)**
Palpitations	1 ( 0.9)	0	1 ( 0.9)
Right ventricular hypertrophy	0	1 ( 1.0)	1 ( 0.9)
Tachycardia	1 ( 0.9)	0	1 ( 0.9)
Angina pectoris	0	1 ( 1.0)	0
Left ventricular hypertrophy	1 ( 0.9)	2 ( 2.0)	0
Myocarditis	0	1 ( 1.0)	0
Pericarditis	1 ( 0.9)	0	0

Table shows all TEAEs reported under the MedDRA System Organ Class *Cardiac Disorders*

### Echocardiographic parameters

Per eligibility criteria, all randomized patients had a LVEF in the normal range at baseline (overall mean, 63.0%, range 50.0–86.0%). LVEF and fractional shortening were relatively stable over the 48 weeks of the trial with no differences between treatment groups in the LS mean change from baseline in either measurement (Table 5). In total, 11 (3.3%) participants had a persistent 10% decline in LVEF during the study, with no significant difference across treatment groups [placebo, 5 (4.3%); tadalafil 0.3 mg/kg, 4 (2.0%), *p* =.45; tadalafil 0.6 mg/kg, 4 (3.6%); *p* = 1.00].

**Table 5:** Summary of echocardiographic left ventricular ejection fraction (LVEF) and shortening fraction

	Placebo (*N* = 116)	Tadalafil 0.3 mg/kg (*N* = 102)	Tadalafil 0.6 mg/kg (*N* = 112)
**LVEF, %**			
Baseline mean (SD)	63.5 (6.4)	63.2 (5.6)	62.4 (6.8)
LS mean change at Week 24 (SE)^a^	-1.0 (0.5)	-1.2 (0.5), *p* =.857	-0.6 (0.5), *p* =.548
LS mean change at Week 48 (SE)^a^	-1.5 (0.6)	-0.3 (0.6), *p* =.127	-0.7 (0.6), *p* =.302
**LV Shortening Fraction**,** %**			
Baseline mean (SD)	35.0 (6.3)	35.5 (5.2)	34.7 (5.5)
LS mean change at Week 24 (SE)^a^	-0.5 (0.4)	-1.1 (0.5), *p* =.307	-0.4 (0.4), *p* =.929
LS mean change at Week 48 (SE)^a^	-0.3 (0.6)	-0.5 (0.6), *p* =.824	0.1 (0.6), *p* =.631

^a^The LS mean change from baseline, standard error, and p-value for the difference between each of the tadalafil groups minus placebo are derived using ANCOVA with factors for treatment and baseline echocardiogram measure as covariate

**Fig. 1:**
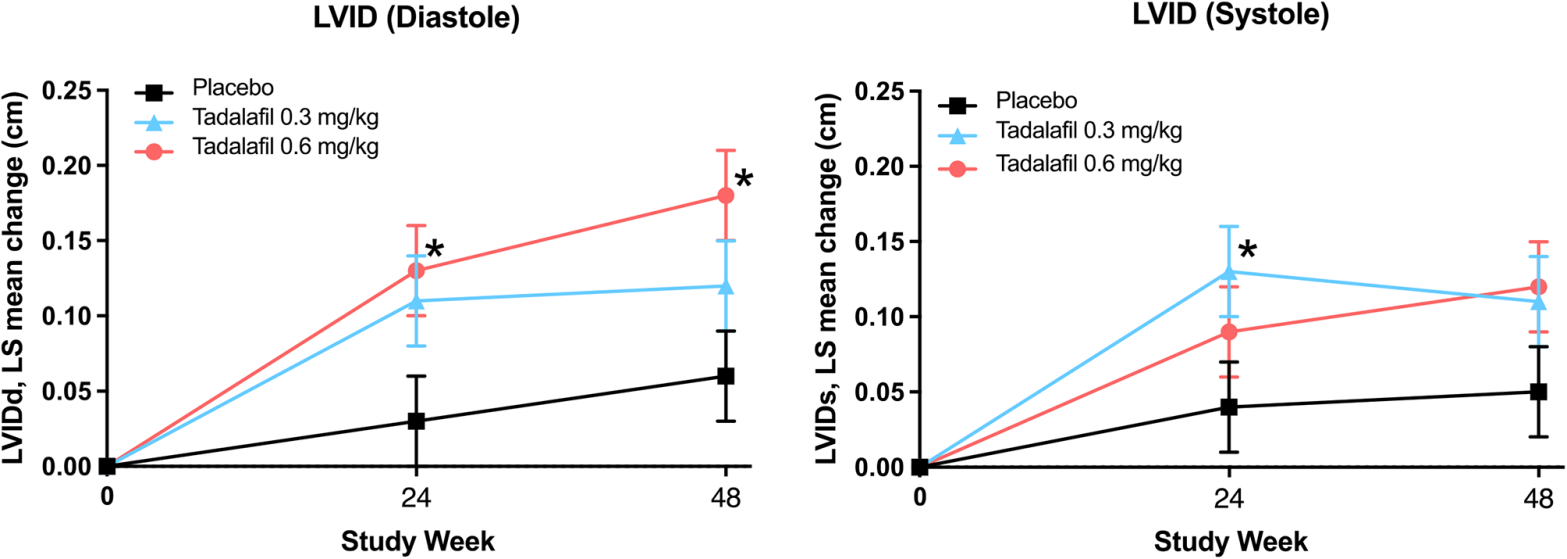
Change from baseline in left ventricular internal dimension (LVID) at diastole (left panel) and systole (right panel) measured by M-mode echocardiography. Points represent the least squares (LS) mean change with standard error. *P <.05 for the difference between each of the tadalafil groups minus placebo are derived using ANCOVA with factors for treatment and baseline echocardiogram measure as covariate

Small mean increases from baseline in diastolic LVID (Fig. 1A) and systolic LVID (Fig. 1B) were observed in both tadalafil treatment groups at Week 24 and Week 48. For diastolic LVID, the LS mean treatment difference between the tadalafil 0.6 mg/kg group and placebo was significant at both Week 24 (+ 0.10 cm, *p* =.019) and Week 48 (+ 0.11 cm, *p* =.008); for systolic LVID, the LS mean treatment difference between the tadalafil 0.3 mg/kg group and placebo was significant at Week 24 (+ 0.09 cm, *p* =.027).

### CMR parameters

A subset of 15 sites in the US collected CMR images at baseline and/or Week 48 from a total of 43 patients. However, because of technical issues, scheduling conflicts, and patient willingness, matching baseline and Week 48 images were only available for 27 patients: 9 in the placebo group, 10 in the tadalafil 0.3 mg/kg group, and 8 in the tadalafil 0.6 mg/kg group.

**Fig. 2:**
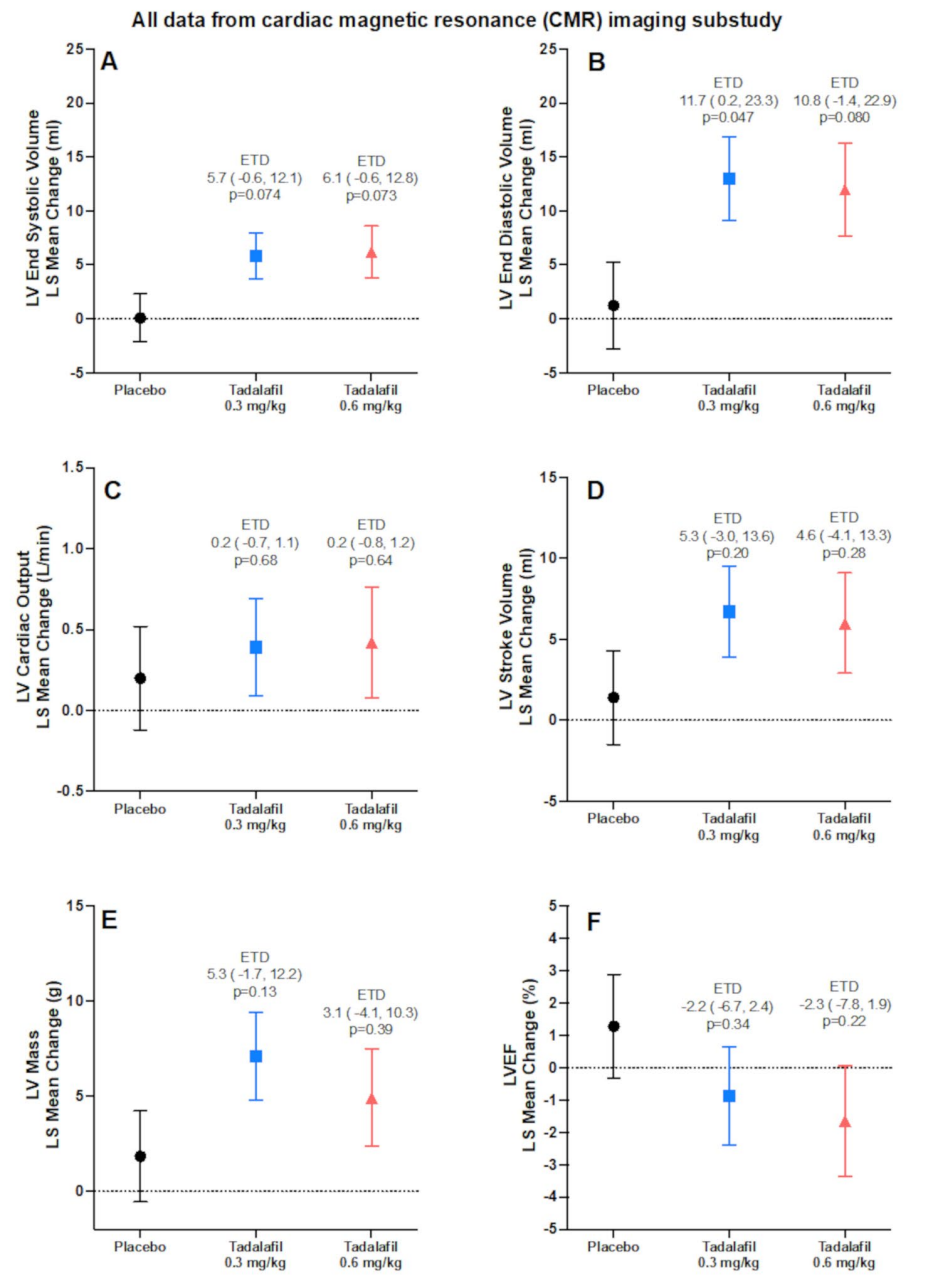
Change from baseline to endpoint (Week 48) or last observation in left ventricular CMR parameters. Points represent the least squares (LS) mean change with standard error. End systolic volume (**A**), end diastolic volume (**B**), cardiac output (**C**), stroke volume (**D**), left ventricular mass (**E**), left ventricular ejection fraction (**F**). ETD = estimated treatment difference for the difference of tadalafil minus placebo with 95% confidence interval from an analysis of covariance (ANCOVA) with change from baseline as dependent variable, baseline value as covariate, and treatment as fixed effect

Mean changes from baseline to endpoint in LV EDV and LV ESV were numerically greater in each tadalafil group compared with placebo, with the mean change in LV EDV significantly greater in the tadalafil 0.3 mg/kg group versus placebo (*p* =.047)(Fig. 2, A and B). Mean changes from baseline to endpoint in cardiac output (Fig. 2C), stroke volume (Fig. 2D), and LV mass (Fig. 2E) also increased to a numerically greater extent in each tadalafil group compared with placebo. Changes in LVEF measured by CMR were small and generally consistent with those measured by echocardiography (Fig. 2F).

Circumferential wall strain (e_cc_) at baseline ranged from − 22.1% to -23.2%, with small changes from baseline to endpoint that were not different between treatment groups [least squares (LS) mean change (SE): placebo, -0.16% (1.4); tadalafil 0.3 mg/kg, 1.0% (1.3)(*p* =.544); tadalafil 0.6 mg/kg, -2.2% (1.5) (*p* =.313)].

### **Analysis of LV** volumes and internal dimension **Z-scores**

Given the consistent pattern for mean increases in each tadalafil group relative to placebo in LVIDs measured by echocardiography and LV volumes measured in a smaller subset by CMR, further analyses were performed to better characterize these changes in context of normal pediatric values for LV volumes and LVIDs, indexed to BSA.

**Fig. 3:**
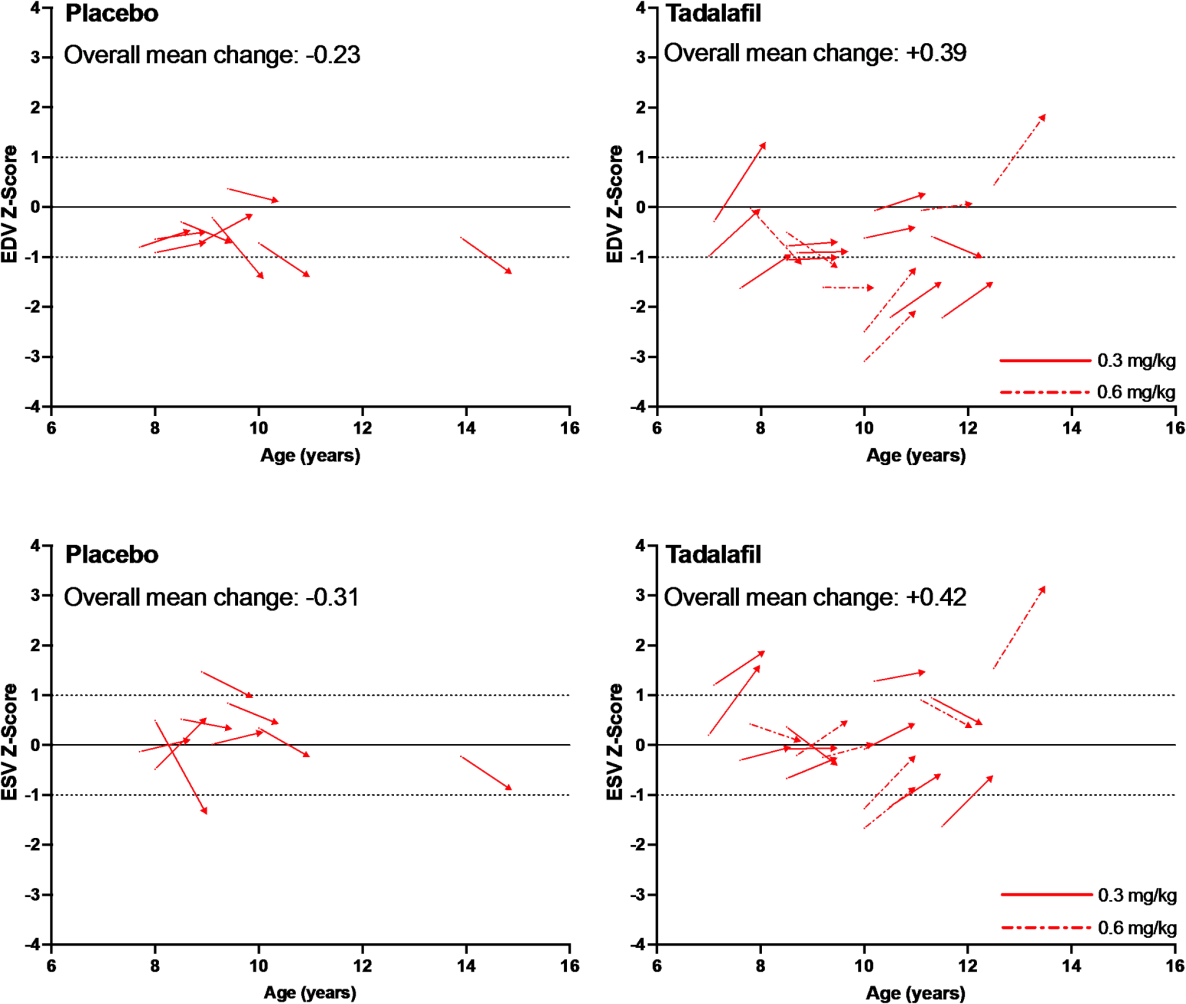
Vector plot of participant-level Z-scores versus age for end diastolic volume (top panels) and end systolic volume (bottom panels) measured by CMR. Each arrow represents an individual participant, with beginning of arrow = baseline Z-score plotted against baseline age (years) and end of arrow = endpoint Z-score (after 48 weeks) plotted against endpoint age

Because of the small sample size, individual patient data for LV volume Z-scores are displayed as vector diagrams with each arrow representing a patient’s age (x-axis) and Z-score (y-axis) at baseline and endpoint for placebo and the combined tadalafil groups (Fig. 3). At baseline, most patient’s Z-scores for EDV in both placebo and tadalafil groups were less than 0, whereas patient’s Z-scores for ESV were distributed more evenly within +/- 1 SD of normal. From baseline to Week 48, the Z-scores for both EDV and ESV declined in most patients in the placebo group, whereas these parameters increased in most patients in the tadalafil groups. Notably, many of the patients in the tadalafil group with increases in ventricular volume, particularly for EDV, had Z-scores less than 0 at baseline that remained less than 0 at 48 weeks, and all but one patient had endpoint volumes within + 2 SD’s of normal.

Table 6 summarizes baseline, endpoint, and change in the Z-scores for diastolic LVID and systolic LVID for the overall study population. Baseline to 48-week changes in Z-scores for diastolic LVID measured by echocardiogram followed generally the same pattern as observed for EDV measured by cMRI: mean Z-scores were negative at baseline for all groups with further decline in the placebo group and but moved toward more normal values in the tadalafil groups at 48 weeks (Table 6). A similar pattern of change was observed for the systolic LVID Z-scores, although the mean baseline value was closer to normal in each of the treatment groups relative to the diastolic LVID Z-score (Table 6).

**Table 6:** Summary of echocardiographic LVID Z-scores

	Placebo (*N* = 116)	Tadalafil 0.3 mg/kg (*N* = 102)	Tadalafil 0.6 mg/kg (*N* = 113)
**LVIDd Z-score**			
n	107	96	100
Baseline mean (SD)	-0.39 (1.4)	-0.54 (1.5)	-0.19 (1.4)
Endpoint mean (SD)	-0.64 (1.4)	-0.51 (1.5)	0.01 (1.5)
LS mean change (SE)^a^	-0.25 (0.1)	-0.03 (0.1)	0.27 (0.1)
LS mean difference vs. placebo^a^		0.22	0.52
95% confidence interval^a^		(-0.09, 0.53)	(0.21, 0.82)
p-value^a^		0.158	0.001
**LVIDs Z-score**			
n	107	95	99
Baseline mean (SD)	-0.18 (1.8)	-0.40 (1.5)	0.03 (1.5)
Endpoint mean (SD)	-0.29 (1.6)	-0.26 (1.6)	0.19 (2.0)
LS mean change (SE)^a^	-0.11 (0.14)	0.06 (0.15)	0.25 (0.14)
LS mean difference vs. placebo^a^		0.17	0.36
95% confidence interval^a^		(-0.23, 0.56)	(-0.04, 0.75)
p-value^a^		0.411	0.076

N: number of patients randomized; n: number of patients with data for the variable; LVIDd: left ventricular internal dimension at diastole; LVIDs, left ventricular internal dimension at systole; SD, standard deviation; SE, standard error; LS, least squares

^a^LS mean, standard error, 2-sided 95% confidence interval and p-value for the difference of tadalafil minus placebo are from an analysis of covariance (ANCOVA) with change from baseline as dependent variable, baseline value as covariate, and treatment as fixed effects

**Fig. 4:**
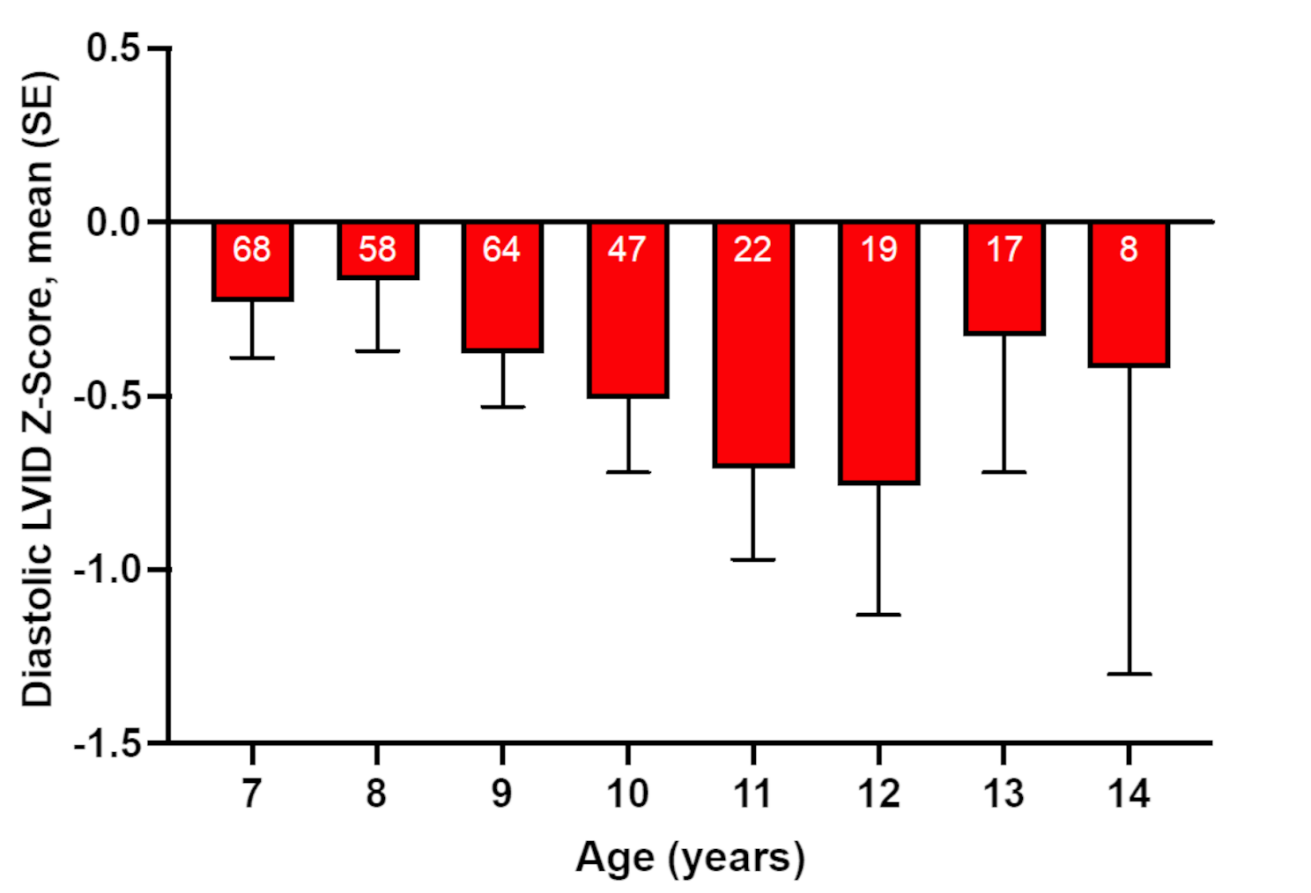
Diastolic left ventricular internal dimension (LVID) Z-score measured by M-mode echocardiography at baseline by age

The finding that the distribution of both LV EDV and diastolic LVID tended to be below the normal range (Z-scores < 0) is consistent with a recent report that the diastolic LVID is smaller than expected values based on age in boys with DMD, particularly between the ages of 7 and 12 years, prior to the onset of clinical cardiomyopathy and LV dilatation [18]. To determine whether a similar pattern was detectable in the present study, mean baseline diastolic LVID Z-scores were plotted for all patients by baseline age category (Fig. 4). The distribution of baseline diastolic LVID by age followed a clear pattern of progressively smaller-than-predicted LV chamber dimensions (greater negative Z-score) especially in the age range of 9–12 years (Fig. 4).

### Effect of Tadalafil on echocardiographic stroke volume and cardiac output in GRMD canine model

**Fig. 5:**
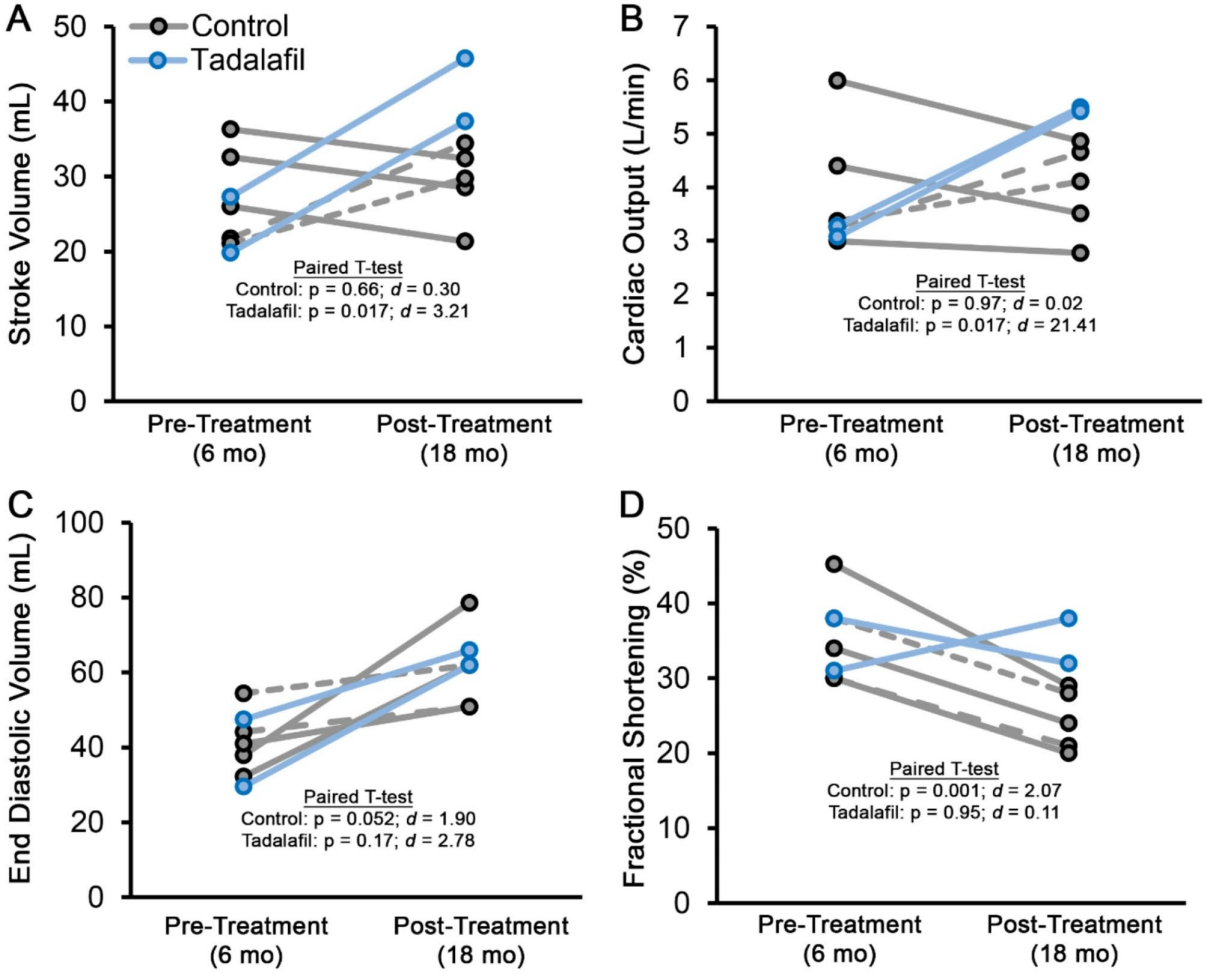
Retrospective analysis on previously published GRMD data. Measures of (**A**) stroke volume, (**B**) cardiac output, (**C**) end diastolic volume, and (**D**) fractional shortening are shown as individual data points for each dog (dashed lines show the individual control dogs that increase SV values with age). Data were analyzed using paired T-tests (α = 0.05). Effect size is shown as Cohen’s d (**d**)

In a previously published study [10], GRMD canines were treated with 1 mg/kg tadalafil as a prophylactic against onset of systolic failure. The dogs began treatment at 9 months of age and reached endpoint at 25 months of age, with tadalafil treatment found to preserve systolic and diastolic LV function [10]. In a retrospective analysis of echocardiography data from this study, individual pre-treatment values (taken at 6 months of age) and post-treatment values (taken at 18 months of age) for stroke volume (SV), cardiac output (CO), EDV, and fractional shortening are plotted for each dog (Fig. 5). While no differences in SV or CO were seen between groups at the pre-treatment age, tadalafil improved both measures at the post-treatment time point, as determined using paired T-tests (Fig. 5A-B). Clear improvements in EDV were not discernable in this retrospective analysis (Fig. 5C), as most control GRMD dogs of this cohort had already started to show onset of dilated cardiomyopathy, including reductions in fractional shortening (Fig. 5D). While the initial intent of this study was to investigate long-term delay of cardiomyopathy by tadalafil treatment, this retrospective data analysis reveals evidence that improvements in diastolic function allow increases in stroke volume and cardiac output to be resolved prior to clear protection of dilated cardiomyopathy by tadalafil treatment in this canine model of DMD. We acknowledge, however, that these data are limited by the small sample size of this study and the lack of echocardiography data acquired at time points immediately preceding and following treatment initiation.

## Discussion

Despite mechanistic and clinical proof-of-concept data, PDE5 inhibition with tadalafil failed to slow the decline in ambulation and mobility in a large Phase 3 trial of boys and young men with DMD 7–14 years of age who were already receiving corticosteroid therapy [17]. This paper reports details of the cardiac safety and CMR sub-study assessments performed during the study.

Tadalafil had no effect, either favorable or adverse, during the 48-week time frame of the study on echocardiographic measures of global cardiac function (LVEF and fractional shortening), ECG qualitative or quantitative changes, vital signs, or cardiac adverse event reporting in boys and young men with DMD. Small increases in LVID on echocardiography in the overall study and LV volumes in a small CMR sub-study were evident in both tadalafil groups compared with placebo. Since global markers of cardiac functioning were unchanged if not slightly improved with tadalafil relative to placebo, the overall clinical relevance and interpretation of these effects of tadalafil on LV chamber dimensions and volumes over the longer term is unclear, particularly given the relatively short time frame of the study.

Global circumferential wall strain measured by CMR is abnormal in DMD patients versus healthy controls prior to the onset of clinically evident declines in LV function, continues to worsen as the disease progresses, and has been proposed as an early marker of LV dysfunction to guide initiation of pharmacological therapy [5, 21]. In this study, circumferential wall strain exhibited little change over the 48 weeks of the study. These findings likely reflect the relatively young age of the study population (mean age 10 years at baseline), the entry criterion which required normal LV function (LVEF > 50%) at baseline, and the relatively short follow up.

The changes in LV volume in each tadalafil group were numerically greater for EDV than ESV, resulting in increases in stroke volume measured by CMR. While observed in the small subset of participants in the CMR study, these changes in LV volume were corroborated by directionally similar changes in echocardiogram-measured LVIDs in all study participants. These findings from the clinical study were also corroborated by the additional analyses of the echocardiogram data from the GRMD animal study.

Increases in LV EDV and LVID associated with improved diastolic function and stroke volume have been reported with PDE5 inhibitors in other patient populations, including heart failure with preserved ejection fraction [22] and diabetic cardiomyopathy [23]. PDE5 inhibition has also been shown to improve indices of diastolic relaxation and function in patients with treatment resistant hypertension [24, 25] and stable systolic heart failure [22]. Plausible pharmacological mechanisms of such improvements have been proposed, including decreased total peripheral resistance, direct effects to improve cardiomyocyte relaxation via local increases in cGMP, and positive remodeling associated with reduced inflammation [25, 26]. The improvements in stroke volume and cardiac output observed in the retrospective assessment of data collected from GRMD canines treated with tadalafil are also directionally consistent with the changes in these parameters measured in the CMR sub-study. Thus, the increases in end diastolic LVID and EDV observed in the current study of boys with DMD are consistent with prior findings and, rather than a spurious finding, may represent a pharmacological consequence of PDE5 inhibition with tadalafil in boys with DMD.

In the context of established dilated cardiomyopathy, increases in LV chamber size is generally considered an adverse finding. Indeed, a study of sildenafil in adults with Duchenne or Becker muscular dystrophy and existing cardiac dysfunction (REVERSE-DMD) was stopped early because of no evidence of benefit, and a numerically higher number of participants experiencing a ≥ 10% increase in ESV while taking sildenafil [11]. Of note, the study population of this prior study was very different from the current study: including smaller sample size (10 subjects per treatment group), all subjects were adults (mean age greater than 20 years) versus pediatric subjects, and all subjects were required to have impaired cardiac function (LVEF < = 45%) at baseline. In addition, the authors of the study highlighted the potential role of reduced cGMP signalling in subjects with established cardiomyopathy as potentially contributing to the observed outcome and also noted pharmacological differences between sildenafil and tadalafil with the former also inhibiting PDE1 possibly producing “off-target side effects.” [11].

On the other hand, impairments in measures of LV relaxation have been characterized in patients with DMD which precede ventricular dilation and clinical cardiomyopathy [27]. More recently, a cross-sectional echocardiographic study of 64 patients suggested that the LV in DMD patients undergoes a period of under-filling or “tonic contraction” prior to the onset of cardiomyopathy and ventricular dilatation [18]. This phenomenon was evidenced by a progressive decline in diastolic LVID Z-score to more negative values beginning around 7 years of age, reaching a nadir around 12 years of age, then reversing to less negative Z-scores around 13 or 14 years of age before further increasing to larger-than-normal values (Z-scores ≥ + 2) in boys 15 years and older as the dilated cardiomyopathy progresses [18]. Cross-sectional analysis of the LVID data by age from the current study further confirmed this phenomenon, displaying an almost identical pattern of progressively more negative diastolic LVID Z-scores in boys between 8 and 12 years of age. The finding that mean Z-scores for both LV EDV and LVID in the placebo group were negative at baseline and further declined over the 48 weeks of follow up further corroborates the hypothesis that younger patients with DMD progress through a period of LV underfilling or “tonic contraction” that phenotypically resembles restrictive cardiomyopathy prior to the onset of LV dilatation and clinical cardiomyopathy.

In this context, the increases in EDV and diastolic LVID with tadalafil in this study were generally from a below-normal range towards more normal values rather than from normal to abnormal values. These effects could potentially reflect improvements in diastolic relaxation and consequent mitigation of the “tonic contraction” phenomenon [18]. Of note, this acute effect of tadalafil appears similar to that reported for ACE inhibitors, where younger (< 15 years) boys with DMD treated prophylactically with ACE inhibitors generally had larger end diastolic LVIDs in a more normal range [18] than those not treated with ACE inhibitors. Nevertheless, the longer-term consequences of such an effect of tadalafil on cardiac function and progression of cardiomyopathy in DMD are unknown. Further study is required to better understand the prognostic value of the “tonic contraction” phenomenon in DMD patients, and whether the longer-term consequences of the pharmacological mitigation of this phenomenon via PDE5 inhibition or other means are favorable with respect to progression of cardiac disease.

Strengths of this study include a large sample size for this rare disease population, randomized placebo-controlled design, and central evaluation of the CMR images. However, the trial was designed to assess the effects of intervention on ambulatory decline rather than progression of cardiac dysfunction. Decline in cardiac function measured by conventional parameters in DMD can be highly variable and generally occurs over a time course longer than the duration of the current study. Moreover, LV function based on global measures was generally normal at baseline with little change over the course of the study, providing limited opportunity to assess any effect of tadalafil. Participation in the CMR sub-study was limited.

## Conclusions

There were no clinically meaningful treatment group differences between tadalafil and placebo in changes in echocardiographic measures of global cardiac function (LVEF and shortening fraction), ECG changes, or reporting of cardiac-related adverse events through 48 weeks in boys with DMD 7 to 14 years of age being treated with corticosteroids. Increases in diastolic LVID measured by echocardiography and LV EDV measured by CMR were evident in both tadalafil groups versus placebo. Compared to age-normative pediatric values for these parameters, these changes were generally small and generally moved from below-normal values toward normative values. Baseline distribution of diastolic LVID Z-scores corroborated the phenomenon of diastolic LV under-filling or “tonic contraction” in younger boys with DMD. Further study of this phenomenon and the longer-term consequences of its pharmacological modification in DMD is warranted.

## Data Availability

Eli Lilly and Company provides access to all individual participant data collected during the trial, after anonymization, with the exception of pharmacokinetic or genetic data. Data are available to request 6 months after the indication studied has been approved in the United States and European Union and after primary publication acceptance, whichever is later. Access is provided after a proposal has been approved by an independent review committee identified for this purpose and after receipt of a signed data sharing agreement. For details on submitting a request, see the instructions provided at www.vivli.org.

## Abbreviations

ACE: Angiotensin converting enzyme
ANCOVA: Analysis of covariance
CMR: Cardiac magnetic resonance
CO: Cardiac output
DMD: Duchenne muscular dystrophy
ECG: Electrocardiogram
EDV: End diastolic volume
GRMD: Golden Retriever muscular dystrophy
LS: Least squares
LV: Left ventricular
LVEF: Left ventricular ejection fraction
LVID: Left ventricular internal dimension
MedDRA: Medical Dictionary for Regulatory Activities
PDE5: Phosphodiesterase-5
SD: Standard deviation
SE: Standard error
SV: Stroke volume
TEAE: Treatment-emergent adverse event

## References

[1] Mavrogeni S, Markousis-Mavrogenis G, Papavasiliou A, Kolovou G. Cardiac involvement in Duchenne and Becker muscular dystrophy. World J Cardiol. 2015;7(7):410–4. 26225202 10.4330/wjc.v7.i7.410 PMC4513493

[2] Buddhe S, Cripe L, Friedland-Little J, Kertesz N, Eghtesady P, Finder J, et al. Cardiac management of the patient with Duchenne muscular dystrophy. Pediatrics. 2018;142(Suppl 2):S72–81. 30275251 10.1542/peds.2018-0333I PMC6566852

[3] Schultz TI, Raucci FJ, Salloum FN. Cardiovascular Disease in Duchenne Muscular Dystrophy: Overview and Insight Into Novel Therapeutic Targets. JACC: Basic to Translational Science. 2022;7(6):608– 25. 10.1016/j.jacbts.2021.11.004 PMC9270569 35818510

[4] Tandon A, Villa CR, Hor KN, Jefferies JL, Gao Z, Towbin JA, et al. Myocardial fibrosis burden predicts left ventricular ejection fraction and is associated with age and steroid treatment duration in Duchenne muscular dystrophy. J Am Heart Association. 2015;4(4):e001338. 10.1161/JAHA.114.001338 PMC4579941 25814625

[5] Hor KN, Wansapura J, Markham LW, Mazur W, Cripe LH, Fleck R, et al. Circumferential strain analysis identifies strata of cardiomyopathy in Duchenne muscular dystrophy: a cardiac magnetic resonance tagging study. J Am Coll Cardiol. 2009;53(14):1204–10. 19341862 10.1016/j.jacc.2008.12.032 PMC2709409

[6] Nigro G, Comi LI, Politano L, Bain RJ. The incidence and evolution of cardiomyopathy in Duchenne muscular dystrophy. Int J Cardiol. 1990;26(3):271–7. 2312196 10.1016/0167-5273(90)90082-g

[7] James K, Gralla J, Ridall L, Do Q, Czaja A, Mourani P, et al. Left ventricular dysfunction in Duchenne muscular dystrophy. Cardiol Young. 2020;30:1–6. 31964455 10.1017/S1047951119002610 PMC8941672

[8] Khairallah M, Khairallah RJ, Young ME, Allen BG, Gillis MA, Danialou G, et al. Sildenafil and cardiomyocyte-specific cGMP signaling prevent cardiomyopathic changes associated with dystrophin deficiency. Proc Natl Acad Sci. 2008;105(19):7028–33. 18474859 10.1073/pnas.0710595105 PMC2383977

[9] Adamo CM, Dai DF, Percival JM, Minami E, Willis MS, Patrucco E, et al. Sildenafil reverses cardiac dysfunction in the Mdx mouse model of Duchenne muscular dystrophy. Proc Natl Acad Sci U S A. 2010;107(44):19079–83. 20956307 10.1073/pnas.1013077107 PMC2973894

[10] Hammers DW, Sleeper MM, Forbes SC, Shima A, Walter GA, Sweeney HL. Tadalafil treatment delays the onset of cardiomyopathy in Dystrophin-Deficient hearts. J Am Heart Association.5(8):e003911. 10.1161/JAHA.116.003911 PMC5015305 27506543

[11] Leung DG, Herzka DA, Thompson WR, He B, Bibat G, Tennekoon G, et al. Sildenafil does not improve cardiomyopathy in Duchenne/Becker muscular dystrophy. Ann Neurol. 2014;76(4):541–9. 25042693 10.1002/ana.24214 PMC4191982

[12] Lu J, Machius M, Dulubova I, Dai H, Südhof TC, Tomchick DR, Rizo J. Primary role of functional ischemia, quantitative evidence for the Two-Hit mechanism, and Phosphodiesterase-5 inhibitor therapy in mouse muscular dystrophy. PLoS ONE. 2007;2. 10.1371/journal.pone.0000806 PMC1950086 17726536

[13] Kobayashi YM, Rader EP, Crawford RW, Iyengar NK, Thedens DR, Faulkner JA, et al. Sarcolemma-localized nNOS is required to maintain activity after mild exercise. Nature. 2008;456(7221):511–5. 18953332 10.1038/nature07414 PMC2588643

[14] Kobayashi YM, Rader EP, Crawford RW, Campbell KP. Endpoint measures in the Mdx mouse relevant for muscular dystrophy pre-clinical studies. Neuromuscul Disord. 2012;22(1):34–42. 22154712 10.1016/j.nmd.2011.08.001 PMC3264796

[15] Percival JM, Whitehead NP, Adams ME, Adamo CM, Beavo JA, Froehner SC. Sildenafil reduces respiratory muscle weakness and fibrosis in the Mdx mouse model of Duchenne muscular dystrophy. J Pathol. 2012;228(1):77–87. 22653783 10.1002/path.4054 PMC4067455

[16] Nelson MD, Rader F, Tang X, Tavyev J, Nelson SF, Miceli MC, et al. PDE5 Inhibition alleviates functional muscle ischemia in boys with Duchenne muscular dystrophy. Neurology. 2014;82(23):2085–91. 24808022 10.1212/WNL.0000000000000498 PMC4118495

[17] Victor RG, Sweeney HL, Finkel R, McDonald CM, Byrne B, Eagle M, et al. A phase 3 randomized placebo-controlled trial of Tadalafil for Duchenne muscular dystrophy. Neurology. 2017;89(17):1811–20. 28972192 10.1212/WNL.0000000000004570 PMC5664308

[18] Su JA, Ramos-Platt L, Menteer J. Left ventricular tonic contraction as a novel biomarker of cardiomyopathy in Duchenne muscular dystrophy. Pediatr Cardiol. 2016;37(4):678–85. 26714815 10.1007/s00246-015-1331-1

[19] Sarikouch S, Peters B, Gutberlet M, Leismann, Birte, Kelter-Kloepping A et al. Sex-Specific Pediatric Percentiles for Ventricular Size and Mass as Reference Values for Cardiac MRI. Circulation: Cardiovascular Imaging. 2010;3(1):65–76. 10.1161/CIRCIMAGING.109.859074 19820203

[20] Bish LT, Sleeper MM, Forbes SC, Wang B, Reynolds C, Singletary GE, et al. Long-term restoration of cardiac dystrophin expression in golden retriever muscular dystrophy following rAAV6-mediated exon skipping. Mol Ther. 2012;20(3):580–9. 22146342 10.1038/mt.2011.264 PMC3293605

[21] Siddiqui S, Alsaied T, Henson SE, Gandhi JP, Patel P, Khoury PR et al. Left Ventricular Magnetic Resonance Imaging Strain Predicts the Onset of Duchenne Muscular Dystrophy–Associated Cardiomyopathy. Circulation: Cardiovascular Imaging. 2020;13:e011526. 10.1161/CIRCIMAGING.120.011526 33190531

[22] Guazzi M, Vicenzi M, Arena R, Guazzi MD. Pulmonary hypertension in heart failure with preserved ejection fraction: a target of phosphodiesterase-5 inhibition in a 1-year study. Circ. 2011;124(2):164–74. 21709061 10.1161/CIRCULATIONAHA.110.983866

[23] Giannetta E, Isidori AM, Galea N, Carbone I, Mandosi E, Vizza CD, et al. Chronic Inhibition of cGMP phosphodiesterase 5A improves diabetic cardiomyopathy. Circulation. 2012;125(19):2323–33. 22496161 10.1161/CIRCULATIONAHA.111.063412

[24] Santos RC, de Faria AP, Barbaro NR, Modolo R, Ferreira-Melo SE, Matos-Souza JR, et al. Tadalafil-induced improvement in left ventricular diastolic function in resistant hypertension. Eur J Clin Pharmacol. 2014;70(2):147–54. 24271647 10.1007/s00228-013-1611-8

[25] Faria AP, Modolo R, Moreno BV, Moreno H. Effects of PDE type 5 inhibitors on left ventricular diastolic dysfunction in resistant hypertension. Arq Bras Cardiol. 2015;104(1):85–9. 25352458 10.5935/abc.20140159 PMC4387615

[26] Storch AS, Velasco LL, Nóbrega A, Gismondi R, Rocha NG. Effects of phosphodiesterase 5 Inhibition on cardiovascular function in resistant hypertension: A systematic review. Life Sci. 2022;291:120270. 34990651 10.1016/j.lfs.2021.120270

[27] Markham LW, Michelfelder EC, Border WL, Khoury PR, Spicer RL, Wong BL, et al. Abnormalities of diastolic function precede dilated cardiomyopathy associated with Duchenne muscular dystrophy. J Am Soc Echocardiogr. 2006;19(7):865–71. 16824995 10.1016/j.echo.2006.02.003

